# Antimicrobial Use in Belgian Acute Care Hospitals : Results of the 2022 ECDC Point Prevalence Survey

**DOI:** 10.1017/ash.2024.328

**Published:** 2024-09-16

**Authors:** Lucy Catteau, Katrien Latour, Morgan Pearcy, Boudewijn Catry

**Affiliations:** Sciensano

## Abstract

**Background:** Point prevalence surveys (PPS) organized by the European Centre for Disease Prevention and Control (ECDC) play a crucial role in assessing healthcare-associated infections (HAIs) and antimicrobial use (AU) in European acute care hospitals. In 2017, a crude prevalence of 28.1% (95% CI 27.3-29.0%) of inpatients receiving at least one antimicrobial was recorded in Belgium (patients ≥65 years: 29.6% (95% CI 28.5-30.7%), < 6 5 years : 26.5% (95%CI 25.3-27.6%)) . Following the challenges posed by the COVID-19 pandemic, the 2022 ECDC-PPS aimed to reassess AU levels. **Method:** A cross-sectional study was conducted between September and November 2022 in 57 representative acute care Belgian hospital sites (35 mergers), following the ECDC-PPS protocol version 6.0. All patients present in surveyed wards at 8 a.m. on the PPS day and not discharged at that time were included. Infection prevention and control teams collected comprehensive data on hospitals, wards, and AU, including agents and indications. **Results:** Among the 10,142 included inpatients, 29.3% (95%CI 28.4-30.2) were receiving at least one antimicrobial (patients ≥65 years: 31.1% (95% CI 29.7-32.4%), < 6 5 years : 27.1% (95%CI 25.6-28.6%)). Intensive care units (56.3%), surgical (38.7%), and medical wards (33.1%) demonstrated the highest AU prevalence, while psychiatric wards exhibited the lowest (3.0%). A total of 3,549 antimicrobials were recorded, commonly prescribed for treating community-acquired infections (48.6%) and HAIs (30.3%, including 4.2% of long-term care facility acquired infections), as well as for surgical and medical prophylaxis (12.4 and 6.6%, respectively). Notably, only 22.7% of surgical prophylaxis courses (n=100/440) lasted more than one day. The top three most used antimicrobial agents consisted of amoxicillin in combination with a beta-lactamase inhibitor (J01CR02, 20.0%), cefazolin (J01DB04, 9.8%) and piperacillin in combination with a beta-lactamase inhibitor (J01CR05, 9.6%). The most frequently reported diagnoses for medical antimicrobial treatment were pneumonia (25.7%) and urinary tract infections (17.1%). The reason for AU was available in 80.0% of the medical notes. **Conclusion:** The 2022 PPS reveals an increased AU prevalence (+1.2%) in Belgian acute care hospitals, especially in patients over 65 years of age (+1.5%). This increase was less pronounced in younger patients (< 6 5y) (+0.6%). Future investigations are crucial to delve into prescription attitudes and modifiable practices, emphasizing the urgent need for robust antimicrobial stewardship programs in these healthcare settings.

